# Deep Learning Estimation of Forced Expiratory Volume in One Second/Forced Vital Capacity and Obstructive Lung Disease Classification From Chest Radiographs With Subgroup Performance Analysis in a North American Cohort: Retrospective Cohort Study

**DOI:** 10.2196/87770

**Published:** 2026-08-05

**Authors:** Eptehal Nashnoush, Helen D'Couto, Benjamin Fine, Leo Anthony Celi, Mohamed Abdalla

**Affiliations:** 1 Trillium Health Centre Mississauga, ON Canada; 2 MedStar Health Washington, DC, MD United States; 3 Department of Medical Imaging Department of Medical Imaging University of Toronto Toronto, ON Canada; 4 Massachusetts Institute of Technology Cambridge, MA United States; 5 Beth Israel Deaconess Medical Center Boston, MA United States; 6 Department of Medicine Faculty of Medicine & Dentistry University of Alberta Edmonton, AB Canada

**Keywords:** deep learning, chest radiography, pulmonary function tests, obstructive lung disease, forced expiratory volume in one second/forced vital capacity ratio, FEV1/FVC ratio

## Abstract

**Background:**

Spirometry is the standard physiological test defining airflow obstruction, the key criterion for diagnosing chronic obstructive pulmonary disease. It is underused in high-income settings and often unavailable in low- and middle-income countries, causing underdetection. Deep learning analysis of chest radiographs, which are widely available where spirometry is not, may complement spirometric screening, but its use in North American cohorts and across demographic strata has not been examined.

**Objective:**

This study aimed to train a deep learning model to estimate the forced expiratory volume in 1 second (FEV₁)/forced vital capacity (FVC) ratio from chest radiographs and classify airflow obstruction (FEV₁/FVC <0.70), evaluate it on a held-out test set, and audit subgroup performance across age, sex, and surname-inferred ethnicity.

**Methods:**

We conducted a retrospective cohort study of 3537 adults who underwent prebronchodilator spirometry and chest radiography within 30 days at a large hospital network in Ontario, Canada, between October 2020 and May 2023. A ConvNeXt-Base architecture pretrained on ImageNet was trained to predict FEV₁/FVC, with predictions classified using a 0.70 cutoff for binary airflow limitation. At the patient level, the cohort was divided into training (n=2263), validation (n=566), and held-out test (n=708 patients; 3273 examinations) sets. Performance was assessed using regression (mean absolute error [MAE], root mean squared error [RMSE], and Pearson r), classification (sensitivity, specificity, positive and negative predictive value [PPV and NPV], and likelihood ratios [LR+ and LR−]), calibration, and decision curve metrics, with 95% CIs from patient-level cluster bootstrap (1000 resamples). Subgroup analyses used Holm correction and two 1-sided tests.

**Results:**

In the held-out test cohort, MAE was 0.08 (95% CI 0.07-0.09) and RMSE was 0.10 (95% CI 0.10-0.11). For binary obstruction, sensitivity was 0.70 (95% CI 0.65-0.74), specificity 0.72 (95% CI 0.67-0.76), PPV 0.71 (95% CI 0.65-0.76), NPV 0.71 (95% CI 0.66-0.76), LR+ 2.46 (95% CI 2.11-2.88), and LR− 0.42 (95% CI 0.36-0.49). Patient-level estimates were similar (sensitivity 0.69, 95% CI 0.66-0.72; specificity 0.74, 95% CI 0.71-0.78). Calibration was excellent for regression (slope=0.97; intercept=0.015) and mildly miscalibrated for the binary task (slope=1.41; intercept=0.04; Brier=0.195). Decision curve analysis showed net benefit at threshold probabilities of approximately 0.27 to 0.86. Sensitivity was meaningfully reduced in Asian patients (0.43, 95% CI 0.29-0.56) compared with White patients (0.75, 95% CI 0.70-0.79; absolute difference −0.32; Holm *P*<.001), with accompanying differences in specificity, PPV, and LR−, and was lower in younger age groups, peaking at 65-74 years.

**Conclusions:**

A deep learning model trained on routine chest radiographs estimated FEV₁/FVC and identified airflow limitation in a North American cohort, with moderate discrimination, well-calibrated regression predictions, and positive net benefit. Performance was not uniform across demographic strata, with reduced sensitivity in Asian patients and younger age groups. Multisite external validation and subgroup-specific verification are important next steps.

## Introduction

### Burden of Obstructive Lung Disease

According to the World Health Organization’s Global Burden of Disease estimates, chronic obstructive pulmonary disease (COPD) was the third leading cause of death worldwide in 2020 [1,2]. Although age-standardized COPD incidence and mortality rates have generally fallen over the past decade, absolute case and death counts have risen as populations age and survive earlier-life respiratory exposures [3]. The Burden of Obstructive Lung Disease study estimated the prevalence of chronic airflow obstruction at 11.2% in men and 8.6% in women, with smoking having the highest mean attributable risk globally [4]. Other risk factors include occupational exposures and outdoor and indoor air pollution [1,2]. The macroeconomic burden of COPD has been estimated at approximately US $4.33 trillion (in 2017 international dollars) from 2020 to 2050 [5].

Estimates of obstructive lung disease burden in resource-limited settings and low- and middle-income countries (LMICs) are limited because of variable and poorly defined risk factors, low awareness of obstructive lung disease, and limited reliability of diagnostic tools [6,7]. In high-income countries, tobacco smoke is the leading cause of COPD. In LMICs, there is growing recognition of the role of indoor air pollution from cooking fuels and lighting sources [2,6], which likely explains the higher incidence of COPD in women in LMICs despite lower smoking rates and the earlier onset of disease resulting from childhood exposure to inhaled pollutants [6].

### Diagnostic Gaps in Spirometry

Airflow obstruction is defined by spirometry: forced vital capacity (FVC) and forced expiratory volume in 1 second (FEV₁) are the 2 measurements used. The American Thoracic Society, European Respiratory Society, and the Global Initiative for Chronic Obstructive Lung Disease (GOLD) recommend identifying obstruction using an FEV₁/FVC ratio below the lower limit of normal or below the GOLD fixed threshold of 0.70 [8]. A clinical diagnosis of COPD requires this spirometric criterion in combination with appropriate clinical context, symptoms, exposure history, and exclusion of alternative diagnoses [8].

The availability and standardization of spirometry vary substantially. In LMICs, spirometry frequently requires calibrated equipment and specialist expertise that are not available, and substitute approaches such as symptom-based questionnaires and peak expiratory flow have historically had limited sensitivity and specificity and lack universally accepted diagnostic thresholds [7]. More recently, machine learning models trained on questionnaire data have shown encouraging discrimination for the long-term risk of airflow obstruction [9], suggesting that data-driven approaches to underserved populations are feasible. Spirometry is also limited by the patient’s ability to perform the test [6,10].

In practice, spirometry remains underused even in high-income settings. A population-based study in Ontario found that only approximately 42% of adults newly diagnosed with asthma underwent pulmonary function testing within the period from 1 year before to 2.5 years after diagnosis [11]. One Ontario regional hospital performs approximately 600 spirometry tests annually [12], whereas more than 20 million diagnostic X-ray procedures are conducted across Canada each year [13]. These figures highlight the scarcity of spirometry relative to the ubiquity of chest radiography and motivate alternative approaches to assess airflow limitation.

### Deep Learning, Chest Imaging, and Prior Work

Quantitative measurements on cross-sectional chest imaging, including emphysema, air trapping, and airway wall thickening on high-resolution computed tomography (CT), have been shown to correlate with FEV₁ and other pulmonary function parameters [14,15]. Deep learning has been applied to thoracic imaging tasks, including detection of pulmonary findings on chest radiographs such as nodule classification [16]. Deep learning has also been used for spirometry quality assurance [17] and structural phenotyping of COPD from spirometric curves [18,19]. More recently, convolutional neural network models have successfully estimated FEV₁ and FVC from low-dose chest CT scans [20-22]. However, CT availability is low in resource-limited and rural settings, and chest radiography is far more prevalent [23,24]. Prior work has shown that deep learning models can estimate spirometric indexes directly from chest radiographs in Japanese cohorts [25]. Whether the underlying radiographic signal is present in other populations and how performance varies across demographic strata remain unclear.

In this study, we train a deep learning model based on a pretrained ConvNeXt architecture to estimate FEV₁/FVC from chest radiographs and classify airflow obstruction; evaluate the resulting model on a held-out test set from a North American cohort; and conduct an exploratory subgroup performance audit across age, sex, and surname-inferred ethnicity. We treat this work as an early exploratory study.

## Methods

### Study Design

We conducted a retrospective cohort study at a large community-based, academically affiliated hospital network in Mississauga, Ontario, Canada, serving a diverse metropolitan catchment area of more than 1 million residents. Eligible patients were adults aged ≥18 years who underwent both a prebronchodilator spirometry test and a chest radiograph within 30 days of each other between October 2020 and May 2023. When multiple pulmonary function tests were available within 30 days of a chest radiograph, only the pair with the shortest time interval was retained. Patients whose chest radiographs were obtained during invasive or noninvasive mechanical ventilation and those with a history of prior lung surgery were excluded. Spirometry reports, including FEV₁ and FVC, were generated by the hospital’s pulmonary function testing system. A total of 3537 unique patients met the inclusion criteria. At the patient level, the cohort was divided into training (n=2263, 64%), validation (n=566, 16%), and held-out test (n=708, 20%) sets, with no overlap of patients across the 3 sets. The held-out test set comprised 3273 examinations from 708 unique patients.

### Ethical Considerations

This retrospective study was approved by the Trillium Health Partners Research Ethics Board (1177). The requirement for individual informed consent was waived because of the retrospective design and the use of deidentified data. This study is reported in accordance with the TRIPOD+AI (Transparent Reporting of a Multivariable Prediction Model for Individual Prognosis or Diagnosis Plus Artificial Intelligence) statement [26]; the completed checklist is provided in Multimedia Appendix 1.

### Data Collection and Preprocessing

Chest radiographs were obtained in DICOM (Digital Imaging and Communications in Medicine) format from the hospital’s imaging system and converted to JPEG for model input. The cohort comprised predominantly posteroanterior views, followed by lateral; left lateral; anteroposterior; and, rarely, right anterior oblique projections. Each radiograph was linked to its paired spirometry report by patient and examination identifiers. FEV₁/FVC was computed from the prebronchodilator FEV₁ and FVC values; examinations with missing or invalid values were excluded.

All images were preprocessed with the PyTorch transformation pipeline. Images were resized to 384×384 pixels to match the ConvNeXt input resolution and normalized using the ImageNet mean (0.485, 0.456, 0.406) and SD (0.229, 0.224, 0.225). Training images were augmented with random rotations up to 15°, random horizontal flips, random affine translations of up to 10% in each direction, random resized cropping with scale 0.9 to 1.1, and random color jitter (brightness and contrast up to 0.2). Validation and held-out test images underwent only deterministic preprocessing (resizing and normalization).

### Model Architecture and Training

We used a ConvNeXt-Base architecture pretrained on ImageNet-22k and subsequently fine-tuned on ImageNet-1k by the original developers [27] at 384×384 input resolution. ConvNeXt-Base was selected as a strong convolutional architecture with state-of-the-art ImageNet performance [27] and well-validated transfer learning behavior on medical imaging tasks [28]. The model was accessed through the Hugging Face Transformers library [29]. The original 1000-class classification head was replaced with an identity layer, and a single fully connected layer with 1 output node was added on top of the resulting 1024-dimensional global feature embedding to predict the continuous FEV₁/FVC ratio.

The model was trained end-to-end with all parameters updateable, using the Adam optimizer (learning rate 1×10⁻⁴), a batch size of 16, and mean squared error loss between the predicted and observed FEV₁/FVC values. A fixed random seed was set to support reproducibility. Training proceeded for 50 epochs on a single NVIDIA V100 graphics processing unit, and the model checkpoint with the lowest validation loss was retained for evaluation on the held-out test set. For the binary airflow obstruction task, predicted FEV₁/FVC values were classified using a 0.70 cutoff, the GOLD fixed criterion for airflow obstruction [8]. The GOLD criterion is defined on postbronchodilator spirometry, whereas this dataset contained prebronchodilator measurements; prebronchodilator spirometry detects somewhat more airflow obstruction but is concordant with postbronchodilator classification in most individuals [30]. The 0.70 threshold reflects this clinical convention applied to the model’s continuous FEV₁/FVC output.

### Statistical Analysis

#### Performance Metrics

Model performance on the held-out test set was characterized using both regression and binary classification metrics. Regression metrics were reported on the FEV₁/FVC scale: mean absolute error (MAE), root mean squared error (RMSE), and Pearson correlation coefficient. Binary classification metrics were derived from thresholding the predicted ratio at 0.70 and included sensitivity, specificity, positive predictive value (PPV), negative predictive value (NPV), *F*_1_-score, positive likelihood ratio (LR+), and negative likelihood ratio (LR−). Likelihood ratios were reported in addition to predictive values because they are prevalence independent, whereas PPV and NPV depend on the test cohort’s obstruction prevalence.

#### Patient-Level Analyses

All 95% CIs were estimated using a patient-level cluster bootstrap with 1000 resamples, with the patient (rather than individual examinations) as the resampling unit to account for within-patient correlation arising from multiple examinations per patient. CIs were reported as the 2.5th and 97.5th percentiles of the bootstrap distribution.

To complement examination-level estimates, a patient-level sensitivity analysis was conducted by randomly selecting 1 examination per patient and recomputing the metrics. This random selection was repeated 100 times, and the mean and 95% interval across selections are reported alongside the examination-level results.

#### Calibration and Decision Curve Analysis

Predicted probabilities of obstruction were obtained by Platt scaling [31] of the predicted FEV₁/FVC ratio. The Platt scaler was fit on the test set; the resulting calibration estimate should therefore be considered internal and modestly optimistic. Binary calibration was summarized by the Brier score, the calibration intercept and slope from a logistic recalibration model, and a calibration plot displaying a locally weighted scatterplot smoothing (loess) curve of observed obstruction frequency against predicted probability across the full range of predictions, with observed frequencies by decile of mean predicted probability overlaid as points with 95% CIs [32]. Regression calibration was additionally summarized by the slope and intercept from a linear regression of observed FEV₁/FVC on model-predicted FEV₁/FVC. Decision curve analysis was conducted over threshold probabilities of 0.05 to 0.95, comparing model net benefit against treat-all and treat-none reference strategies.

#### Subgroup Comparisons

An exploratory subgroup performance audit characterized differences across sex, age group, and surname-inferred ethnicity. Age was stratified into 18 to 44, 45 to 54, 55 to 64, 65 to 74, 75 to 84, and >85 years. The 18- to 24-years and 25- to 44-years age groups were pooled into a single 18- to 44-years stratum because airflow obstruction is uncommon in younger adults, and major epidemiological studies of obstructive lung disease similarly restrict or aggregate younger age groups for this reason [4].

In the absence of self-identified race and ethnicity in the dataset, ethnicity was inferred from patient surnames as a proxy using the averaging-of-proportions method proposed by Abdalla et al [33], which combines US Census surname data (2000 and 2010) to assign each surname a probability distribution over broad racial or ethnic categories. Each surname was matched to the census surname lists directly or by closest spelling match and assigned to its most probable category (White, Asian, Black, or Hispanic); surnames absent from the census reference data even after approximate matching were grouped as unknown and reported descriptively but excluded from formal pairwise comparisons. Subgroup results based on this proxy are exploratory.

Within each grouping variable, every nonreference level was compared against a prespecified reference level. Reference levels were the largest stratum within each grouping variable: female for sex, 65 to 74 years for age, and White for ethnicity. For each metric, the difference between the nonreference and reference level was estimated using a paired patient-level bootstrap with 1000 resamples, with 2-sided *P* values derived from the bootstrap distribution of the difference. Equivalence was assessed using two 1-sided tests (TOST) against a prespecified absolute-difference margin of ±0.05, with a pair classified as equivalent when the 95% bootstrap interval for the difference fell entirely within that margin. Within each grouping variable and metric, *P* values were adjusted for multiple comparisons using Holm step-down procedure.

## Results

### Overall Classification Performance

The held-out test cohort comprised 708 patients (379 female patients and 329 male patients) contributing 3273 examinations, with an obstruction prevalence of 49.4% (1618/3273). For the continuous FEV₁/FVC prediction, the model achieved a MAE of 0.08 (95% CI 0.07-0.09), a RMSE of 0.10 (95% CI 0.10-0.11), and a Pearson correlation of 0.60 (95% CI 0.54-0.65). For the binary obstruction task at the 0.70 threshold (Table 1), the model achieved a sensitivity of 0.70 (95% CI 0.65-0.74), specificity of 0.72 (95% CI 0.67-0.76), PPV of 0.71 (95% CI 0.65-0.76), NPV of 0.71 (95% CI 0.66-0.76), and F1 score of 0.70 (95% CI 0.66-0.74). Likelihood ratios were LR+ 2.46 (95% CI 2.11-2.88) and LR− 0.42 (95% CI 0.36-0.49). Patient-level estimates closely matched the examination-level estimates (sensitivity 0.69, 95% CI 0.66-0.72; specificity 0.74, 95% CI 0.71-0.78; PPV 0.68, 95% CI 0.65-0.71; NPV 0.75, 95% CI 0.73-0.77; Table 2).

**Table 1:** Confusion matrix for binary obstruction classification^a^.

True class	Model prediction	
	Predicted: no obstruction	Predicted: obstruction
No obstruction (n=1655, 50.6%), n	1186	469
Obstruction (n=1618, 49.4%), n	489	1129
Total (n=3273), n (%)	1675 (51.2)	1598 (48.8)

^a^Cells show counts of test-set examinations. Row percentages indicate the proportion of true-class examinations classified into each predicted class. Column percentages indicate the proportion of predicted-class examinations belonging to each true class.

**Table 2:** Overall performance on the held-out test cohort.

Metric	Examination level, estimate (95% CI)	Patient level, estimate (95% CI)
Sensitivity	0.70 (0.65-0.74)	0.69 (0.66-0.72)
Specificity	0.72 (0.67-0.76)	0.74 (0.71-0.78)
PPV^a^	0.71 (0.65-0.76)	0.68 (0.65-0.71)
NPV^b^	0.71 (0.66-0.76)	0.75 (0.73-0.77)
*F*_1_-score	0.70 (0.66-0.74)	0.69 (0.66-0.71)
LR+^c^	2.46 (2.11-2.88)	2.69 (2.36-3.08)
LR−^d^	0.42 (0.36-0.49)	0.42 (0.38-0.46)
MAE^e^	0.08 (0.07-0.09)	0.08 (0.07-0.08)
RMSE^f^	0.10 (0.10-0.11)	0.10 (0.10-0.10)
Pearson *r*	0.60 (0.54-0.65)	0.60 (0.55-0.64)

^a^PPV: positive predictive value.

^b^NPV: negative predictive value.

^c^LR+: positive likelihood ratio.

^d^LR–: negative likelihood ratio.

^e^MAE: mean absolute error.

^f^RMSE: root mean squared error.

### Calibration and Decision Curve Analysis

**Figure 1:**
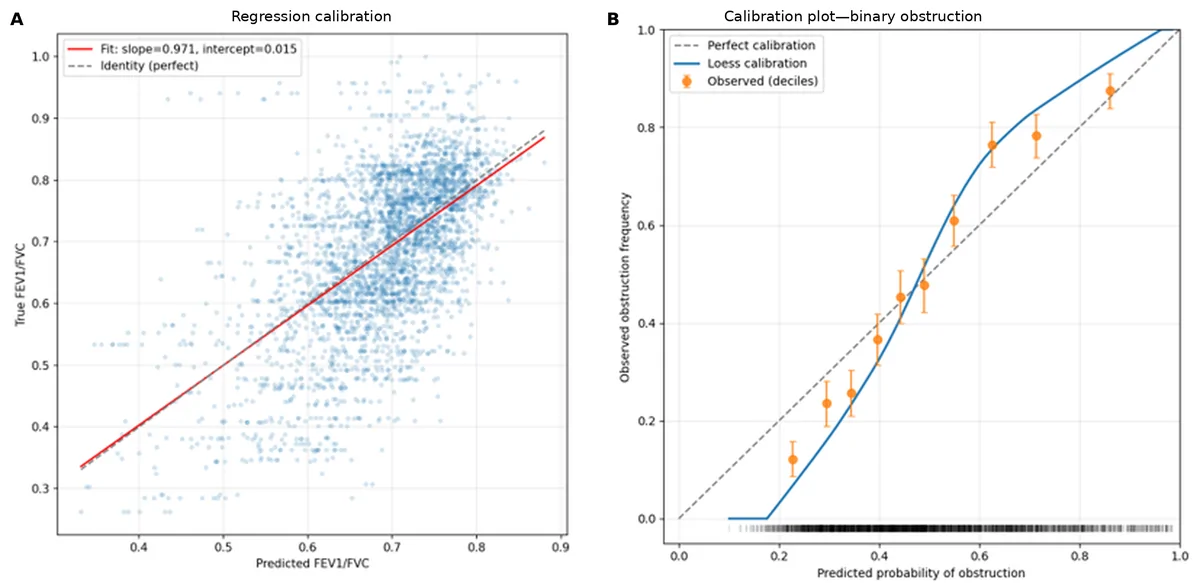
Calibration of the forced expiratory volume in 1 second (FEV₁)/forced vital capacity (FVC) and binary obstruction predictions on the held-out test cohort. (A) Regression calibration: scatter of observed against predicted FEV₁/FVC, with the identity line shown for reference. (B) Binary obstruction calibration: observed obstruction frequency plotted against predicted probability of obstruction. The solid line is a loess-smoothed calibration curve fitted across the full range of predicted probabilities; points show observed frequencies by decile of mean predicted probability with 95% CIs. The dashed identity line indicates perfect calibration.

Regression calibration was excellent, with a slope of 0.97 and an intercept of 0.015 estimated from the linear regression of observed vs predicted FEV₁/FVC values (Figure 1A). Binary probability calibration showed mild miscalibration (Brier score=0.195; calibration intercept=0.04; calibration slope=1.41; Figure 1B), with the loess calibration curve indicating slight overprediction at low predicted probabilities and underprediction at high predicted probabilities.

**Figure 2:**
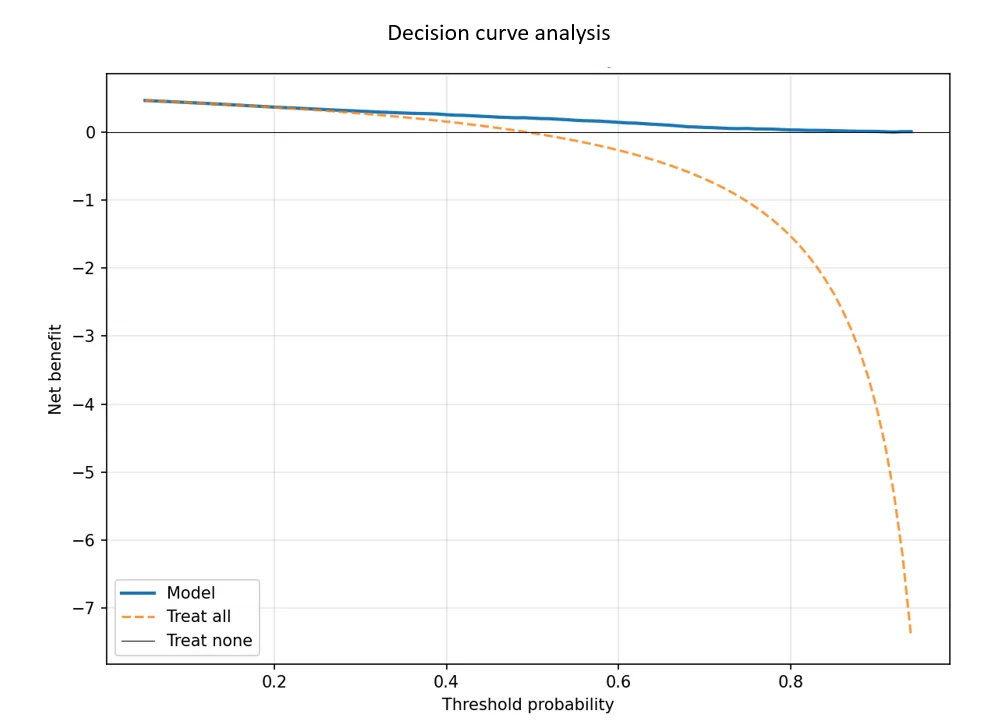
Decision curve analysis on the held-out test cohort.

Decision curve analysis over threshold probabilities of 0.05 to 0.95 showed positive net benefit relative to both treat-all and treat-none strategies across the threshold range of 0.08 to 0.94 (Figure 2). However, the incremental net benefit over the treat-all strategy was minimal at threshold probabilities below approximately 0.27, and above approximately 0.86 the model curve approached the treat-none strategy. At a net-benefit margin of 0.02, improvement over both reference strategies was concentrated in the intermediate threshold range of approximately 0.27 to 0.86.

### Subgroup Performance

#### Overview

Subgroup performance was characterized across sex, age group, and surname-inferred ethnicity using paired patient-level bootstrap with 2-sided *P* values and Holm correction within each grouping variable and metric. Equivalence at a ±0.05 margin was assessed using TOST. The full pairwise comparison matrix is provided in Multimedia Appendix 2. Of the 54 comparisons, 13 reached statistical significance after Holm correction. No comparison met the ±0.05 equivalence criterion, which requires the 95% CI of the difference to fall entirely within that margin.

#### Performance by Sex

Performance estimates by sex are reported in Table 3. Sensitivity was higher among male patients (0.74, 95% CI 0.68-0.79) than among female patients (0.65, 95% CI 0.59-0.71), with the pairwise difference (absolute difference +0.08, 95% CI−0.00 to +0.17) borderline nonsignificant after Holm correction (Holm *P*=.052). PPV was significantly higher in male patients (absolute difference +0.13, 95% CI +0.03 to +0.23, Holm *P*=.02), consistent with the higher obstruction prevalence in this subgroup (898/1583, 56.7% vs 720/1690, 42.6%). Differences in specificity, NPV, and likelihood ratios did not reach statistical significance.

**Table 3:** Performance by sex and surname-inferred ethnicity.

Subgroup			Examinations, N		Prevalence, n (%)		Sensitivity (95% CI)		Specificity (95% CI)		PPV^a^ (95% CI)		NPV^b^ (95% CI)		LR+^c^ (95% CI)		LR−^d^ (95% CI)
**Sex**																	
	Female	1690		720 (42.6)		0.65 (0.59-0.71)		0.72 (0.67-0.77)		0.64 (0.54-0.72)		0.73 (0.67-0.80)		2.33 (1.92-2.93)		0.48 (0.39-0.58)	
	Male	1583		898 (56.7)		0.74 (0.68-0.79)		0.71 (0.65-0.77)		0.77 (0.70-0.83)		0.67 (0.60-0.74)		2.54 (2.07-3.17)		0.37 (0.30-0.46)	
**Surname-inferred ethnicity**																	
	White (reference)	2054		1232 (60)		0.75 (0.70-0.79)		0.67 (0.62-0.73)		0.77 (0.72-0.82)		0.64 (0.57-0.71)		2.26 (1.93-2.75)		0.38 (0.31-0.46)	
	Asian	616		191 (31)		0.43 (0.29-0.56)		0.81 (0.74-0.88)		0.51 (0.31-0.67)		0.76 (0.65-0.86)		2.29 (1.38-3.70)		0.70 (0.54-0.89)	
	Hispanic	172		45 (26.2)		0.68 (0.43-0.91)		0.64 (0.50-0.83)		0.40 (0.16-0.70)		0.85 (0.69-0.97)		1.90 (1.11-4.16)		0.50 (0.13-0.92)	
	Black	107		51 (47.7)		0.82 (0.52-1.00)		0.82 (0.65-0.97)		0.81 (0.36-0.97)		0.84 (0.60-1.00)		4.61 (2.06-15.95)		0.22 (0.00-0.60)	
	Unknown	318		99 (31.1)		0.57 (0.40-0.74)		0.73 (0.63-0.82)		0.48 (0.29-0.68)		0.79 (0.67-0.89)		2.07 (1.34-3.31)		0.60 (0.36-0.84)	

^a^PPV: positive predictive value.

^b^NPV: negative predictive value.

^c^LR+: positive likelihood ratio.

^d^LR−: negative likelihood ratio.

#### Performance by Age Group

Performance estimates by age group are reported in Table 4. Sensitivity was significantly lower in the 18-44 year stratum (0.30, 95% CI 0.15-0.46) than in the 65-to-74-year reference (0.76, 95% CI 0.69-0.82; absolute difference −0.46; Holm *P*<.001) and in the 45-to-54-year stratum (0.46, 95% CI 0.25-0.64; absolute difference −0.32; Holm *P*=.008). Specificity was significantly higher in both younger strata (18 to 44 years: absolute difference +0.32; Holm *P*<.001; 45 to 54 years: absolute difference +0.26; Holm *P*<.001), and NPV was significantly higher (18 to 44 years: absolute difference +0.22; Holm *P*=.03; 45 to 54 years: absolute difference +0.20; Holm *P*=.03). LR− was significantly worse in the 18-to-44-year stratum (absolute difference +0.36; Holm *P*=.01). Performance in the 55-to-64-year, 75-to-84-year, and >85-year age groups did not differ significantly from the 65-to-74-year reference group on any metric after Holm correction.

**Table 4:** Performance by age group.

Age group (years)	Exams, N	Prevalence, n (%)	Sensitivity (95% CI)	Specificity (95% CI)	PPV^a^ (95% CI)	NPV^b^ (95% CI)	LR+^c^ (95% CI)	LR−^d^ (95% CI)
18-44	303	55 (18.2)	0.30 (0.15-0.46)	0.92 (0.88-0.96)	0.46 (0.21-0.73)	0.86 (0.77-0.93)	3.88 (1.70-9.53)	0.76 (0.58-0.93)
45-54	310	71 (22.9)	0.46 (0.25-0.64)	0.86 (0.79-0.92)	0.49 (0.22-0.72)	0.85 (0.76-0.92)	3.33 (1.65-6.29)	0.63 (0.42-0.88)
55-64	622	255 (41)	0.68 (0.57-0.78)	0.74 (0.65-0.82)	0.65 (0.49-0.78)	0.77 (0.68-0.85)	2.62 (1.87-3.87)	0.43 (0.29-0.61)
65-74 (reference)	924	536 (58)	0.76 (0.69-0.82)	0.60 (0.51-0.69)	0.72 (0.62-0.81)	0.64 (0.52-0.76)	1.90 (1.51-2.52)	0.40 (0.28-0.55)
75-84	850	535 (62.9)	0.73 (0.67-0.80)	0.63 (0.54-0.71)	0.77 (0.68-0.84)	0.58 (0.46-0.69)	1.97 (1.59-2.56)	0.43 (0.31-0.55)
>85	264	166 (62.9)	0.65 (0.49-0.87)	0.49 (0.34-0.66)	0.68 (0.48-0.85)	0.45 (0.23-0.79)	1.26 (0.85-1.99)	0.73 (0.29-1.26)

^a^PPV: positive predictive value.

^b^NPV: negative predictive value.

^c^LR+: positive likelihood ratio.

^d^LR−: negative likelihood ratio.

#### Performance by Surname-Inferred Ethnicity

Performance estimates by surname-inferred ethnicity are reported in Table 3. Asian patients (n=616 examinations) showed a multimetric pattern of conservative prediction relative to White patients: sensitivity was significantly lower (0.43 vs 0.75; absolute difference −0.32; Holm *P*<.001), specificity significantly higher (0.81 vs 0.67; absolute difference +0.14; Holm *P*=.006), PPV significantly lower (0.51 vs 0.77; absolute difference −0.26; Holm *P*=.01), and LR− significantly worse (absolute difference +0.33; Holm *P*<.001). NPV did not differ significantly. Hispanic patients (n=172 examinations) showed significantly higher NPV than White patients (0.85 vs 0.64; absolute difference +0.21; Holm *P*=.048), consistent with lower obstruction prevalence in this subgroup (45/172, 26.2% vs 1232/2054, 60%). Sensitivity, specificity, PPV, and likelihood ratios did not differ significantly. Black vs White comparisons (n=107 examinations in the Black subgroup) were statistically inconclusive on all metrics, with wide CIs reflecting the small subgroup size.

## Discussion

### Principal Findings

This study provides early exploratory evidence that a deep learning model can extract signals predictive of FEV₁/FVC and airflow obstruction from routine chest radiographs in a North American cohort. The model showed moderate discrimination, well-calibrated regression predictions, mild miscalibration in the binary task, and positive decision curve net benefit, with improvements over treat-all and treat-none reference strategies concentrated in the intermediate threshold range of approximately 0.27 to 0.86.

Performance was not uniform across demographic strata. The patient-level cluster bootstrap with Holm correction identified meaningfully reduced sensitivity in Asian patients relative to White patients (absolute difference −0.32; Holm *P*<.001), paired with higher specificity (+0.14; Holm *P*=.006), lower PPV (−0.26; Holm *P*=.01), and worse LR− (+0.33; Holm *P*<.001). This pattern is consistent with the model operating more conservatively in this subgroup, where the observed obstruction prevalence was also lower. Substantial age-related variation was documented: sensitivity was significantly lower in the 18- to 44-years (−0.46; Holm *P*<.001) and 45- to 54-years (−0.32; Holm *P*=.008) age groups relative to the 65 to 74 years reference, paired with significantly higher specificity in both younger strata. This again is a conservative pattern in subgroups with lower observed obstruction prevalence. A statistically significant difference in NPV between Hispanic and White patients (+0.21; Holm *P*=.048) was also identified; given NPV’s prevalence dependence and the lower obstruction prevalence in the Hispanic subgroup, this finding likely reflects prevalence rather than differential discrimination. Sex differences in sensitivity were borderline nonsignificant after correction (+0.08; Holm *P*=.052); PPV was significantly higher in male patients (+0.13; Holm *P*=.02), consistent with higher obstruction prevalence in the male subgroup.

### Limitations

The study has several limitations that should be acknowledged. The retrospective single‑center design means that findings may not fully generalize without further external validation, which we have not yet performed. All performance estimates were derived from a single random training, validation, or test partition; although the patient-level cluster bootstrap quantifies metric uncertainty conditional on this split, performance may vary under alternative partitions. Ethnicity was inferred from patient surnames using a census‑derived proxy; this approach does not capture self‑identified race, ethnicity, or social context and carries some risk of misclassification. Subgroup sample sizes vary across strata; in particular, the Black subgroup (n=107 examinations) was small, and the Black vs White comparison was statistically inconclusive on all metrics. Equivalence at a ±0.05 margin (TOST) was not established for any subgroup pair, reflecting the precision limit of the test cohort rather than signaling broad demographic disparities. Turning to calibration, binary probability calibration showed mild miscalibration (slope=1.41), with predicted probabilities slightly compressed toward the middle of the range. Regression calibration of the underlying continuous prediction was excellent (slope=0.97; intercept=0.015), suggesting that the binary miscalibration arises from the thresholding step rather than from systematic bias in the regression. The 0.70 threshold was additionally applied to prebronchodilator spirometry rather than the postbronchodilator measurement in the GOLD definition; because prebronchodilator testing identifies more obstruction, the model may overidentify obstruction relative to a postbronchodilator reference standard [30].

### Future Directions

Several directions for future work emerge naturally from these findings. Multisite external validation in independent North American and international cohorts is the highest-priority next step, both to assess generalizability and to provide an opportunity to recalibrate binary predictions in cohorts with representative disease prevalence. Prospective evaluation with self-identified demographic data would address the surname-proxy limitation and enable subgroup-specific net benefit to be formally characterized. Additionally, integrating clinical metadata such as smoking history and comorbidities could further improve model performance. The present study used a single ConvNeXt-Base architecture, which does not establish optimal predictive performance for this task. Systematic comparisons of alternative architectures, as well as ensemble and stacking approaches, may yield meaningful differences in performance and represent a natural direction for future investigation.

### Conclusions

This study adds to a growing body of evidence that chest radiographs carry signals predictive of pulmonary function that can be extracted by deep learning models. In a North American cohort, the model achieved moderate discrimination, well-calibrated regression predictions, and positive decision curve net benefit, but performance varied across demographic strata, particularly in Asian patients and younger age groups. Multisite external validation and subgroup-specific performance verification are needed to establish whether these findings generalize beyond the present cohort.

## Data Availability

The data supporting this study consist of linked clinical and imaging records from Trillium Health Partners and cannot be made publicly available due to patient privacy regulations and institutional data governance policies. Researchers interested in replication may contact the corresponding author to discuss access through the Trillium Health Partners Research Ethics Board. The analysis code and trained model weights are available from the corresponding author upon request. The model was implemented in Python using PyTorch and the Hugging Face Transformers library; the ConvNeXt-Base architecture is publicly available on Hugging Face [29].

## Appendix

Multimedia Appendix 1 TRIPOD+AI checklist.

Multimedia Appendix 2 Pairwise subgroup comparisons with Holm-corrected *P* values and outcomes of two one-sided tests for equivalence.

## Abbreviations

COPD: chronic obstructive pulmonary disease
CT: computed tomography
DICOM: Digital Imaging and Communications in Medicine
FEV1: forced expiratory volume in 1 second
FVC: forced vital capacity
GOLD: Global Initiative for Chronic Obstructive Lung Disease
LMIC: low- and middle-income country
LR–: negative likelihood ratio
LR+: positive likelihood ratio
MAE: mean absolute error
NPV: negative predictive value
PPV: positive predictive value
RMSE: root mean squared error
TOST: two 1-sided tests
TRIPOD+AI: Transparent Reporting of a Multivariable Prediction Model for Individual Prognosis or Diagnosis Plus Artificial Intelligence
