# Supplementary material for: Deep Learning Estimation of Forced Expiratory Volume in One Second/Forced Vital Capacity and Obstructive Lung Disease Classification From Chest Radiographs With Subgroup Performance Analysis in a North American Cohort: Retrospective Cohort Study
Source: JMIR AI. 2026 Aug 5;5:e87770. doi: 10.2196/87770 (PMC13490935; doi:10.2196/87770)
Supplement: Supplementary file 1 [file ai_v5i1e87770_app1.pdf]

Multimedia Appendix 1. TRIPOD+AI Checklist

Reference: Collins GS, Moons KGM, Dhiman P, et al. TRIPOD+AI statement: updated guidance for reporting clinical prediction models that use regression or machine learning methods. *BMJ*. 2024;385:e078378. doi:10.1136/bmj-2023-078378

Manuscript: Deep-Learning Estimation of FEV_1/FVC and Obstructive Lung Disease Classification From Chest Radiographs With Subgroup Performance Analysis in a North American Cohort: A Retrospective Cohort Study.

D = model development only; E = model evaluation/validation only; D;E = applicable to both.

Section / Topic	Item	D / E	Checklist Item (verbatim — Collins et al. BMJ 2024)	Location in Manuscript
**TITLE AND ABSTRACT**				
Title	**1**	D;E	Identify the study as developing or evaluating the performance of a multivariable prediction model, the target population, and the outcome to be predicted.	Title page. Deep-Learning Estimation of FEV_1/FVC and Obstructive Lung Disease Classification From Chest Radiographs With Subgroup Performance Analysis in a North American Cohort: A Retrospective Cohort Study. Study type (retrospective cohort), target population (North American adults), and outcome (FEV_1/FVC / airflow obstruction) all identified.
Abstract	**2**	D;E	See TRIPOD+AI for Abstracts checklist.	Structured abstract (Background, Objective, Methods, Results, Conclusions). Reports sample sizes, key metrics with 95% CIs, subgroup findings, and conclusions.
**INTRODUCTION**				
Background	**3a**	D;E	Explain the healthcare context (including whether diagnostic or prognostic) and rationale for developing or evaluating the prediction model, including references to existing models.	Introduction Burden of obstructive lung disease; §Diagnostic gaps in spirometry; §Deep learning, chest imaging, and prior work. Diagnostic context (FEV_1/FVC estimation from CXR). Prior work in Japanese cohorts cited (Ueda et al. 2024 [18]).
	**3b**	D;E	Describe the target population and the intended purpose of the prediction model in the context of the care pathway, including its intended users (e.g., healthcare professionals, patients, public).	Introduction (final paragraph). Target population: adults undergoing chest radiography. Purpose: complement to spirometric screening where spirometry is unavailable. Intended users – N/A
	**3c**	D;E	Describe any known health inequalities between sociodemographic groups.	Addressed in the introduction. Sex-related COPD burden differences and LMIC disparities mentioned.
Objectives	**4**	D;E	Specify the study objectives, including whether the study describes the development or validation of a prediction model (or both).	Introduction (final paragraph). Objectives explicitly stated: train a deep-learning model...evaluate the resulting model...conduct an exploratory subgroup performance audit. Both development and internal evaluation stated.
**METHODS**				

Section / Topic	Item	D / E	Checklist Item (verbatim — Collins et al. BMJ 2024)	Location in Manuscript
Data	**5a**	D;E	Describe the sources of data separately for the development and evaluation datasets (e.g., randomised trial, cohort, routine care or registry data), the rationale for using these data, and representativeness of the data.	Methods §Study Design and Ethics. Retrospective routine-care cohort, Trillium Health Partners, Ontario, Canada. Same source for development and evaluation (patient-level partition from same dataset). Rationale: availability of linked paired spirometry and CXR. Representativeness: diverse metropolitan catchment of >1 million residents stated.
	**5b**	D;E	Specify the dates of the collected participant data, including start and end of participant accrual; and, if applicable, end of follow-up.	Methods §Study Design and Ethics. October 2020 and May 2023. Cross-sectional; no follow-up.
Participants	**6a**	D;E	Specify key elements of the study setting (e.g., primary care, secondary care, general population) including the number and location of centres.	Methods §Study Design and Ethics. Large community-based, academically affiliated hospital network in Mississauga, Ontario, Canada (Trillium Health Partners). Single-centre.
	**6b**	D;E	Describe the eligibility criteria for study participants.	Methods §Study Design and Ethics. Inclusion: adults ≥18 yrs with pre-bronchodilator spirometry + CXR within 30 days (Oct 2020–May 2023). Exclusion: CXR during invasive/non-invasive mechanical ventilation; prior lung surgery.
	**6c**	D;E	Give details of any treatments received, and how they were handled during model development or evaluation, if relevant.	Methods §Study Design and Ethics. Pre-bronchodilator spirometry specified throughout. Only pre-bronchodilator measurements used; bronchodilator administration therefore not a confounder.
Data preparation	**7**	D;E	Describe any data pre-processing and quality checking, including whether this was similar across relevant sociodemographic groups.	Methods §Data Collection and Preprocessing. DICOM → JPEG; resize 384×384; ImageNet normalization (μ/σ). Training augmentation described (rotations, flips, affine, crop, colour jitter). Test: deterministic only.
Outcome	**8a**	D;E	Clearly define the outcome that is being predicted and the time horizon, including how and when assessed, the rationale for choosing this outcome, and whether the method of outcome assessment is consistent across sociodemographic groups.	Methods §Study Design and Ethics; §Statistical Analysis. Outcome 1: continuous FEV_1/FVC. Outcome 2: binary airflow obstruction (FEV_1/FVC < 0.70; GOLD fixed threshold). Pre-bronchodilator spirometry from hospital PFT system. Threshold rationale cited (GOLD/ATS/ERS, Introduction). Automated measurement → consistent across groups.
	**8b**	D;E	If outcome assessment requires subjective interpretation, describe the qualifications and demographic characteristics of the outcome assessors.	Not applicable. Spirometry is automated and objective; generated by hospital pulmonary-function testing system. No subjective interpretation required.
	**8c**	D;E	Report any actions to blind assessment of the outcome to be predicted.	Methods §Data Collection and Preprocessing. Spirometry collected independently of and prior to CXR model pipeline. Automated outcome; blinding not required. Retrospective design ensures no outcome contamination of predictor collection.

Section / Topic	Item	D / E	Checklist Item (verbatim — Collins et al. BMJ 2024)	Location in Manuscript
Predictors	**9a**	D	Describe the choice of initial predictors (e.g., literature, previous models, all available predictors) and any pre-selection of predictors before model building.	Methods §Data Collection and Preprocessing; §Model Architecture and Training. Single input: raw chest radiograph image (end-to-end CNN; no hand-crafted features). Predictor choice motivated by prior literature showing CXR contains spirometry-predictive signal (ref [18]). No predictor pre-selection — feature extraction entirely learned.
	**9b**	D;E	Clearly define all predictors, including how and when they were measured (and any actions to blind assessment of predictors for the outcome and other predictors).	Methods §Data Collection and Preprocessing. Predictor: chest radiograph (DICOM, converted to JPEG, resized 384×384). Obtained within 30 days of spirometry. Projection types reported (PA predominant). Automated pipeline with no human radiograph interpretation — blinding is structurally guaranteed.
	**9c**	D;E	If predictor measurement requires subjective interpretation, describe the qualifications and demographic characteristics of the predictor assessors.	Not applicable. Radiographs fed directly into model pipeline without any human subjective interpretation.
Sample size	**10**	D;E	Explain how the study size was arrived at (separately for development and evaluation), and justify that the study size was sufficient to answer the research question. Include details of any sample size calculation.	Methods §Study Design and Ethics. All eligible patients used (n = 3,537 total; test n = 708 patients / 3,273 examinations.
Missing data	**11**	D;E	Describe how missing data were handled. Provide reasons for omitting any data.	Methods §Data Collection and Preprocessing. Examinations with missing or invalid [FEV_1/FVC] values were excluded. Complete-case analysis; no imputation.
Analytical methods	**12a**	D	Describe how the data were used (e.g., for development and evaluation of model performance) in the analysis, including whether the data were partitioned, considering any sample size requirements.	Methods §Study Design and Ethics. Patient-level 70/15/15 partition: training (n=2,263), validation (n=566), held-out test (n=708). Ensuring that no patient contributed to more than one split, data leakage explicitly prevented. Sample size requirements for partition not formally discussed.
	**12b**	D	Depending on the type of model, describe how predictors were handled in the analyses (functional form, rescaling, transformation, or any standardisation).	Methods §Data Collection and Preprocessing. Images resized to 384×384; normalised using ImageNet mean (0.485, 0.456, 0.406) and SD (0.229, 0.224, 0.225). Full augmentation pipeline described for training.
	**12c**	D	Specify the type of model, rationale, all model-building steps, including any hyperparameter tuning, and method for internal validation.	Methods §Model Architecture and Training. ConvNeXt-Base; pretrained ImageNet-22k/1k; rationale stated (strong ImageNet performance; validated medical imaging transfer-learning). End-to-end fine-tuning; Adam (lr = 1×10^{-4}); MSE loss; batch 16; 50 epochs; best validation-loss checkpoint retained. Fixed random seed. No formal hyperparameter search beyond learning rate.

Section / Topic	Item	D / E	Checklist Item (verbatim — Collins et al. BMJ 2024)	Location in Manuscript
	**12d**	D;E	Describe if and how any heterogeneity in estimates of model parameter values and model performance was handled and quantified across clusters (e.g., hospitals, countries). See TRIPOD-Cluster for additional considerations.	Single-centre study; no multi-centre clustering present. Not applicable. Subgroup heterogeneity in model performance (sex, age, ethnicity) addressed under items 14 and 23a.
	**12e**	D;E	Specify all measures and plots used (and their rationale) to evaluate model performance (e.g., discrimination, calibration, clinical utility) and, if relevant, to compare multiple models.	Methods §Statistical Analysis. Regression: MAE, RMSE, Pearson r. Classification: sensitivity, specificity, PPV, NPV, F1, LR+, LR−. Rationale for LR± explicitly stated (prevalence-independence). Calibration: Brier score, intercept, slope, decile calibration plot. Clinical utility: decision-curve analysis (treat-all / treat-none comparators). Single model — no multi-model comparison needed.
	**12f**	E	Describe any model updating (e.g., recalibration) arising from the model evaluation, either overall or for particular sociodemographic groups or settings.	Methods §Calibration. Platt scaling applied to generate predicted obstruction probabilities; scaler fit on held-out test set. Noted as 'internal and modestly optimistic.' No subgroup-specific recalibration performed.
	**12g**	E	For model evaluation, describe how the model predictions were calculated (e.g., formula, code, object, application programming interface).	Methods §Model Architecture and Training; §Statistical Analysis. Model trained and evaluated within this study. Predictions generated by forward pass through trained ConvNeXt-Base → continuous FEV_1/FVC → threshold at 0.70.
Class imbalance	**13**	D;E	If class imbalance methods were used, state why and how this was done, and any subsequent methods to recalibrate the model or the model predictions.	Obstruction prevalence was ~49.4% in the test set (approximately balanced).
Fairness	**14**	D;E	Describe any approaches that were used to address model fairness and their rationale.	Methods §Subgroup comparisons. Comprehensive subgroup performance audit across sex, age band (18–44, 45–54, 55–64, 65–74, 75–84, 85+), and surname-inferred ethnicity (White, Asian, Black, Hispanic, Unknown). Paired patient-level bootstrap with Holm-corrected pairwise comparisons; TOST equivalence tests (±0.05 margin). Surname proxy method (Abdalla et al. [20]) described and acknowledged.
Model output	**15**	D	Specify the output of the prediction model (e.g., probabilities, classification). Provide details and rationale for any classification and how the thresholds were identified.	Methods §Study Design and Ethics; §Statistical Analysis. Output 1: continuous FEV_1/FVC ratio. Output 2: binary classification via threshold at 0.70 (GOLD fixed criterion; ATS/ERS). Threshold choice referenced in Introduction.
Training vs. evaluation	**16**	D;E	Identify any differences between the development and evaluation data in healthcare setting, eligibility criteria, outcome, and predictors.	Methods §Study Design and Ethics. Development and evaluation data are from the same single-centre retrospective cohort, partitioned at the patient level. No differences in setting, eligibility, outcome, or predictors exist.

Section / Topic	Item	D / E	Checklist Item (verbatim — Collins et al. BMJ 2024)	Location in Manuscript
Ethical approval	**17**	D;E	Name the institutional research board or ethics committee that approved the study and describe the participant-informed consent or the ethics committee waiver of informed consent.	Methods §Study Design and Ethics. This retrospective study was approved by the Trillium Health Partners Research Ethics Board (REB 1177). The requirement for individual informed consent was waived given the retrospective design and use of de-identified data.
**OPEN SCIENCE**				
Funding	**18a**	D;E	Give the source of funding and the role of the funders for the present study.	Acknowledgments. This research received no external funding.
Conflicts of interest	**18b**	D;E	Declare any conflicts of interest and financial disclosures for all authors.	Conflicts of Interest section. None declared.
Protocol	**18c**	D;E	Indicate where the study protocol can be accessed or state that a protocol was not prepared.	A study protocol was prepared as part of the Research Ethics Board application (REB 1177, Trillium Health Partners). The protocol is not publicly available but may be requested from the corresponding author.
Registration	**18d**	D;E	Provide registration information for the study, including register name and registration number, or state that the study was not registered.	See 18d
Data sharing	**18e**	D;E	Provide details of the availability of the study data.	The data supporting this study consist of linked clinical and imaging records from Trillium Health Partners and cannot be made publicly available due to patient privacy regulations and institutional data governance policies. Researchers interested in replication may apply for access through the Trillium Health Partners Research Ethics Board.
Code sharing	**18f**	D;E	Provide details of the availability of the analytical code.	See code and model availability section in the paper
**PATIENT & PUBLIC INVOLVEMENT**				
Patient & Public Involvement	**19**	D;E	Provide details of any patient and public involvement during the design, conduct, reporting, interpretation, or dissemination of the study or state no involvement.	N/A
**RESULTS**				
Participants	**20a**	D;E	Describe the flow of participants through the study, including the number of participants with and without the outcome and, if applicable, a summary of the follow-up time. A diagram may be helpful.	Methods §Study Design and Ethics. Total eligible n = 3,537; training 2,263; validation 566; test 708 patients / 3,273 examinations. Exclusion criteria stated.
	**20b**	D;E	Report the characteristics overall and, where applicable, for each data source or setting, including the key dates, key predictors (including demographics), treatments received, sample size, number of outcome events, follow-up time, and amount of missing data. A table may be helpful. Report any differences across key demographic groups.	Results §Overall classification performance; Tables II–IV. Test set: 708 patients (379F, 329M), 3,273 examinations, obstruction prevalence 49.4% (1,618/3,273). Subgroup

Section / Topic	Item	D / E	Checklist Item (verbatim — Collins et al. BMJ 2024)	Location in Manuscript
				characteristics (sex, ethnicity, age band) with prevalence reported
	**20c**	E	For model evaluation, show a comparison with the development data of the distribution of important predictors (demographics, predictors, and outcome).	Not provided.
Model development	**21**	D;E	Specify the number of participants and outcome events in each analysis (e.g., for model development, hyperparameter tuning, model evaluation).	Methods §Study Design and Ethics; Results §Overall classification performance. Test: 708 patients, 3,273 examinations, 1,618 obstructed (49.4%). Training n = 2,263; validation n = 566 stated.
Model specification	**22**	D	Provide details of the full prediction model (e.g., formula, code, object, API) to allow predictions in new individuals and to enable third-party evaluation and implementation, including any restrictions to access or re-use (e.g., freely available, proprietary).	Methods §Model Architecture and Training & Code and Model Availability section. Architecture fully described (ConvNeXt-Base; Hugging Face model ID cited [21].
Model performance	**23a**	D;E	Report model performance estimates with confidence intervals, including for any key subgroups (e.g., sociodemographic). Consider plots to aid presentation.	Results §Overall classification performance (Table I: examination- and patient-level with 95% CIs); §Subgroup performance (Tables II–III with 95% CIs); §Calibration and decision-curve analysis (Figures 1–2). All metrics with 95% CIs from patient-level cluster bootstrap (1,000 resamples). Fully reported.
	**23b**	D;E	If examined, report results of any heterogeneity in model performance across clusters. See TRIPOD Cluster for additional details.	Single-centre study; no multi-centre clustering. Not applicable.
Model updating	**24**	E	Report the results from any model updating, including the updated model and subsequent performance.	Results §Calibration. Platt scaling results reported: slope 1.41, intercept 0.04, Brier 0.195. Post-calibration performance noted.
**DISCUSSION**				
Interpretation	**25**	D;E	Give an overall interpretation of the main results, including issues of fairness in the context of the objectives and previous studies.	Discussion §Principal findings. Results interpreted relative to Ueda et al. 2024 (Japanese cohorts). Fairness/subgroup findings discussed in detail (Asian patients, younger age bands, Hispanic NPV, sex). Cautious framing as 'early, exploratory evidence.'
Limitations	**26**	D;E	Discuss any limitations of the study (such as a non-representative sample, sample size, overfitting, missing data) and their effects on any biases, statistical uncertainty, and generalizability.	Discussion §Limitations. Covers: retrospective single-centre design; surname-proxy ethnicity (not self-identified); small Black subgroup (n = 107 exams); TOST equivalence not established (precision limit); Platt scaling optimism (scaler fit on test set); no external validation.
Usability	**27a**	D	Describe how poor quality or unavailable input data (e.g., predictor values) should be assessed and handled when implementing the prediction model.	N/A

Section / Topic	Item	D / E	Checklist Item (verbatim — Collins et al. BMJ 2024)	Location in Manuscript
	**27b**	D	Specify whether users will be required to interact in the handling of the input data or use of the model, and what level of expertise is required of users.	N/A
	**27c**	D;E	Discuss any next steps for future research, with a specific view to applicability and generalizability of the model.	Discussion §Future directions; Conclusion. Multi-site external validation; prospective evaluation with self-identified demographics; clinical metadata integration (smoking, comorbidities); CNN–transformer architecture comparisons.

Abbreviations: ATS = American Thoracic Society; CXR = chest radiograph; DCA = decision-curve analysis; ERS = European Respiratory Society; FEV_1 = forced expiratory volume in 1 second; FVC = forced vital capacity; GOLD = Global Initiative for Chronic Obstructive Lung Disease; LR = likelihood ratio; MAE = mean absolute error; NPV = negative predictive value; PFT = pulmonary function test; PPI = patient and public involvement; PPV = positive predictive value; RMSE = root-mean-squared error; TOST = two one-sided tests.
