# Supplementary material for: Deep Learning Estimation of Forced Expiratory Volume in One Second/Forced Vital Capacity and Obstructive Lung Disease Classification From Chest Radiographs With Subgroup Performance Analysis in a North American Cohort: Retrospective Cohort Study
Source: JMIR AI. 2026 Aug 5;5:e87770. doi: 10.2196/87770 (PMC13490935; doi:10.2196/87770)
Supplement: Supplementary file 2 [file ai_v5i1e87770_app2.pdf]

Multimedia Appendix 2. Pairwise subgroup comparisons with Holm-corrected P-values and TOST equivalence outcomes

This appendix reports all 54 pairwise comparisons between non-reference subgroup levels and their respective reference (sex: female; age: 65-74 years; ethnicity: White). For each comparison, the absolute difference (Δ) in the metric is reported with its 95% confidence, the two-sided P-value, the Holm-corrected P-value, and whether the comparison met the TOST equivalence criterion at a ±0.05 margin. Rows with Holm-corrected P < .05 are shaded for visibility. Across all 54 comparisons, 13 reached statistical significance after Holm correction.

Sex comparisons (reference: Female)

Comparison	Metric	Δ	95% CI of Δ	P (2-sided)	P (Holm)	Equivalent at ±0.05?
Male vs Female	Sensitivity	+0.08	(-0.00, +0.17)	.052	.052	No
Male vs Female	Specificity	-0.01	(-0.10, +0.07)	.772	.772	No
Male vs Female	PPV	**+0.13**	(+0.03, +0.23)	.020	**.020**	No
Male vs Female	NPV	-0.06	(-0.17, +0.05)	.246	.246	No
Male vs Female	LR+	+0.20	(-0.59, +0.97)	.594	.594	No
Male vs Female	LR−	-0.11	(-0.23, +0.01)	.096	.096	No

Age band comparisons (reference: 65–74 years)

Comparison	Metric	Δ	95% CI of Δ	P (2-sided)	P (Holm)	Equivalent at ±0.05?
55-64 vs 65-74	Sensitivity	-0.08	(-0.21, +0.05)	.218	.654	No
55-64 vs 65-74	Specificity	+0.14	(+0.01, +0.27)	.032	.096	No
55-64 vs 65-74	PPV	-0.08	(-0.26, +0.10)	.404	>.99	No
55-64 vs 65-74	NPV	+0.13	(-0.02, +0.27)	.086	.258	No
55-64 vs 65-74	LR+	+0.77	(-0.26, +2.04)	.160	.550	No
55-64 vs 65-74	LR−	+0.03	(-0.17, +0.24)	.784	>.99	No
75-84 vs 65-74	Sensitivity	-0.03	(-0.12, +0.07)	.564	.672	No
75-84 vs 65-74	Specificity	+0.03	(-0.10, +0.15)	.674	.674	No
75-84 vs 65-74	PPV	+0.05	(-0.08, +0.17)	.458	>.99	No
75-84 vs 65-74	NPV	-0.06	(-0.22, +0.10)	.446	.540	No
75-84 vs 65-74	LR+	+0.07	(-0.69, +0.79)	.836	.836	No
75-84 vs 65-74	LR−	+0.02	(-0.16, +0.19)	.754	>.99	No
85+ vs 65-74	Sensitivity	-0.10	(-0.30, +0.11)	.336	.672	No
85+ vs 65-74	Specificity	-0.11	(-0.28, +0.07)	.216	.432	No
85+ vs 65-74	PPV	-0.05	(-0.25, +0.14)	.662	>.99	No
85+ vs 65-74	NPV	-0.17	(-0.42, +0.18)	.270	.540	No

Comparison	Metric	Δ	95% CI of Δ	P (2-sided)	P (Holm)	Equivalent at ±0.05?
85+ vs 65-74	LR+	-0.59	(-1.30, +0.26)	.110	.550	No
85+ vs 65-74	LR−	+0.30	(-0.16, +0.83)	.196	.588	No
18-44 vs 65-74	Sensitivity	**-0.46**	(-0.63, -0.28)	<.001	**<.001**	No
18-44 vs 65-74	Specificity	**+0.32**	(+0.22, +0.43)	<.001	**<.001**	No
18-44 vs 65-74	PPV	-0.26	(-0.55, +0.03)	.086	.344	No
18-44 vs 65-74	NPV	**+0.22**	(+0.07, +0.36)	.006	**.030**	No
18-44 vs 65-74	LR+	+2.49	(-0.41, +7.97)	.116	.550	No
18-44 vs 65-74	LR−	**+0.36**	(+0.14, +0.57)	.002	**.010**	No
45-54 vs 65-74	Sensitivity	**-0.32**	(-0.52, -0.10)	.002	**.008**	No
45-54 vs 65-74	Specificity	**+0.26**	(+0.15, +0.37)	<.001	**<.001**	No
45-54 vs 65-74	PPV	-0.25	(-0.50, +0.01)	.064	.320	No
45-54 vs 65-74	NPV	**+0.20**	(+0.06, +0.34)	.008	**.032**	No
45-54 vs 65-74	LR+	+1.48	(-0.40, +4.27)	.164	.550	No
45-54 vs 65-74	LR−	+0.24	(-0.02, +0.50)	.088	.352	No

Ethnicity comparisons (reference: White)

Comparison	Metric	Δ	95% CI of Δ	P (2-sided)	P (Holm)	Equivalent at ±0.05?
Asian vs White	Sensitivity	**-0.32**	(-0.46, -0.18)	<.001	**<.001**	No
Asian vs White	Specificity	**+0.14**	(+0.05, +0.23)	.002	**.006**	No
Asian vs White	PPV	**-0.26**	(-0.45, -0.07)	.004	**.012**	No
Asian vs White	NPV	+0.11	(-0.03, +0.23)	.104	.208	No
Asian vs White	LR+	+0.09	(-1.01, +1.66)	.966	>.99	No
Asian vs White	LR−	**+0.33**	(+0.14, +0.51)	<.001	**<.001**	No
Black vs White	Sensitivity	+0.06	(-0.23, +0.24)	.390	.780	No
Black vs White	Specificity	+0.15	(-0.01, +0.30)	.076	.152	No
Black vs White	PPV	+0.00	(-0.39, +0.21)	.826	.826	No
Black vs White	NPV	+0.19	(-0.08, +0.37)	.142	.208	No
Black vs White	LR+	+3.36	(-0.14, +13.67)	.081	.242	No
Black vs White	LR−	-0.14	(-0.37, +0.22)	.238	.476	No
Hispanic vs White	Sensitivity	-0.06	(-0.31, +0.18)	.652	.780	No
Hispanic vs White	Specificity	-0.02	(-0.19, +0.19)	.798	.798	No

Comparison	Metric	Δ	95% CI of Δ	P (2-sided)	P (Holm)	Equivalent at ±0.05?
Hispanic vs White	PPV	-0.36	(-0.61, -0.02)	.040	.080	No
Hispanic vs White	NPV	**+0.21**	(+0.05, +0.35)	.016	**.048**	No
Hispanic vs White	LR+	-0.07	(-1.34, +2.51)	.652	>.99	No
Hispanic vs White	LR−	+0.11	(-0.27, +0.56)	.606	.606	No
